# Optimization of γ-Aminobutyric Acid Production by *Lactobacillus plantarum* Taj-Apis362from Honeybees

**DOI:** 10.3390/molecules20046654

**Published:** 2015-04-15

**Authors:** Naser Tajabadi, Afshin Ebrahimpour, Ali Baradaran, Raha Abdul Rahim, Nor Ainy Mahyudin, Mohd Yazid Abdul Manap, Fatimah Abu Bakar, Nazamid Saari

**Affiliations:** 1 Department of Food Science, Faculty of Food Science and Technology, University Putra Malaysia, Serdang 43400, Selangor, Malaysia; E-Mails: ntajabadi@yahoo.com (N.T.); afshin.ebrahimpour@gmail.com (A.E.); norainy@upm.edu.my (N.A.M.); fatim@upm.edu.my (F.A.B.); 2 Department of Honey Bee, Animal Science Research Institute of Iran (ASRI), Karaj 315851483, Iran; 3 Departments of Cell and Molecular Biology, Faculty of Biotechnology and Biomolecular Sciences, University Putra Malaysia, Serdang 43400, Selangor, Malaysia; E-Mails: alib_100@yahoo.com (A.B.); raha@upm.edu.my (R.A.R.); 4 Department of Food Technology, Faculty of Food Science and Technology, University Putra Malaysia, Serdang 43400, Selangor, Malaysia; E-Mail: myazid@upm.edu.my

**Keywords:** γ-aminobutyric acid (GABA), response surface methodology (RSM), glutamic acid, *Lactobacillus plantarum* Taj-Apis362

## Abstract

Dominant strains of lactic acid bacteria (LAB) isolated from honey bees were evaluated for their γ-aminobutyric acid (GABA)-producing ability. Out of 24 strains, strain Taj-Apis362 showed the highest GABA-producing ability (1.76 mM) in MRS broth containing 50 mM initial glutamic acid cultured for 60 h. Effects of fermentation parameters, including initial glutamic acid level, culture temperature, initial pH and incubation time on GABA production were investigated via a single parameter optimization strategy. The optimal fermentation condition for GABA production was modeled using response surface methodology (RSM). The results showed that the culture temperature was the most significant factor for GABA production. The optimum conditions for maximum GABA production by *Lactobacillus plantarum* Taj-Apis362 were an initial glutamic acid concentration of 497.97 mM, culture temperature of 36 °C, initial pH of 5.31 and incubation time of 60 h, which produced 7.15 mM of GABA. The value is comparable with the predicted value of 7.21 mM.

## 1. Introduction

γ-Aminobutyric acid (GABA) is a non-protein amino acid biosynthesized by glutamic acid decarboxylase (GAD), a pyridoxal-5'-phosphate-dependent enzyme, which catalyzes the irreversible α-decarboxylation of L-glutamic acid to GABA. γ-Aminobutyric acid is known as one of the major inhibitory neurotransmitters in the sympathetic nervous system, exerting antihypertensive and anti-diabetic effects in humans [1]. In addition, GABA can control lipid levels in serum, as well as pain and anxiety [2]. Moreover, consumption of GABA-enriched foods inhibits cancer cell proliferation [3]. Hence, GABA has been viewed as a bioactive component in pharmaceuticals and foods [4].

γ-Aminobutyric acid production by various micro-organisms such as fungi, yeasts and lactic acid bacteria (LAB) have been reported [3,4,5]. Among the microbes, LAB are of interest to the food industry as they are generally regarded as safe (GRAS) organisms. Several GABA-producing lactobacilli have been reported, such as *Lactobacillus senmaizukei* isolated from traditional pickles in Japan [6], *Lactococcus lactis* obtained from cheese in Japan [7], *Lactobacillus paracasei* isolated from cheese in Italy [8] and Japanese traditional fermented fish [9], *Lactobacillus brevis* isolated from Kimchi in Japan [10] and South Korea [3] and *Lactobacillus delbrueckii* subsp. *bulgaricus* [8]. In the present study, we evaluated the GABA-producing ability of *Lactobacillus* strains which had been isolated from the honey stomach and honeycombs of the honeybee *Apis dorsata* in Malaysia [11,12]. Evaluation for different GABA-producing LABs is important for the food industry because individual LAB have specific fermentation profiles, such as flavor formation and acid-producing ability [13].

Different fermentation factors affect the rate of GABA production by microorganisms. Among these factors the most common and essential ones are incubation time, initial pH, incubation temperature and initial glutamic acid concentration [14]. The fermentation conditions can be optimized using single-variable-at-a-time and response surface methodology (RSM) based on the GAD activity of the fermenting microorganisms. The most significant stages in the biological process are modeling and optimization to improve a system and increase the efficiency of the process. At the optimum pH 5.0, the highest GABA production was achieved by *L. brevis* [15]. Similarly, the glutamate content 500 mM in the culture medium increased GABA by optimizing the fermentation condition of *L. paracasei* NFRI 7415 at pH 5.0 [9]. The GABA production by *Streptococcus salivarius* subsp*. thermophilus* Y2 was also enhanced by optimizing the fermentation conditions at pH 4.5 [16]. *Lactobacillus* *brevis* GABA 100 fermenting black raspberry juice produced maximum GABA levels at pH 3.5 and 30 °C on the 12th day of fermentation [4]. In addition, the GABA production by *L. brevis* was enhanced by optimizing fermentation conditions at an initial pH of 5.25 and 37 °C [17]. Therefore, the optimum conditions vary among the fermenting microorganisms due to the different properties of the GADs. In the current study, a single variable optimization design used as the first step was efficient for identifying which ranges of fermentation factors had a significant effect on the GABA production. Then response surface methodology was used to optimize the fermentative parameter for the high production of GABA. Therefore, the aim of this study was to evaluate GABA-producing LABs from the honey stomach and honeycomb of honeybees, and to optimize the fermentation conditions for maximum GABA production using the best isolate. 

## 2. Results and Discussion

### 2.1. Evaluation of GABA-Producing Lactic Acid Bacteria

**Figure 1:**
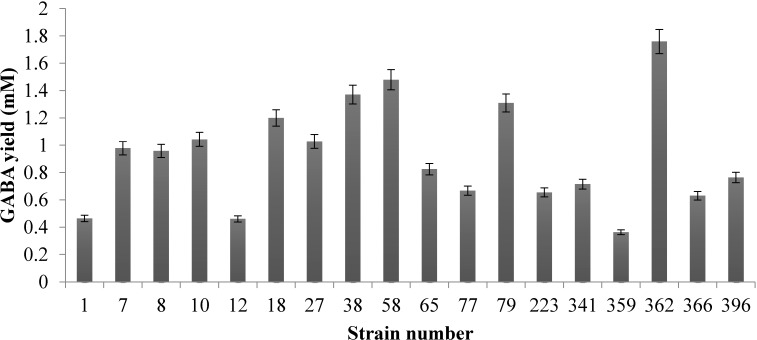
Comparison of GABA production by 18 LAB strains isolated from honeycomb and honey stomach of honeybees. The strains were cultivated in MRS broth containing 50 mM initial glutamic acid at 30 °C for 60 h. The GABA content in the supernatants was analyzed by HPLC method as described. Data are expressed as mean ± SD from triplicate experiments.

In this study, a total of 24 dominant LAB strains isolated in our previous study were evaluated for their GABA-producing ability. Among them, 18 strains were able to produce GABA (Figure 1), among which *L. plantarum* Taj-Apis362 showed the highest GABA production (1.76 mM) as measured using HPLC.

**Figure 2:**
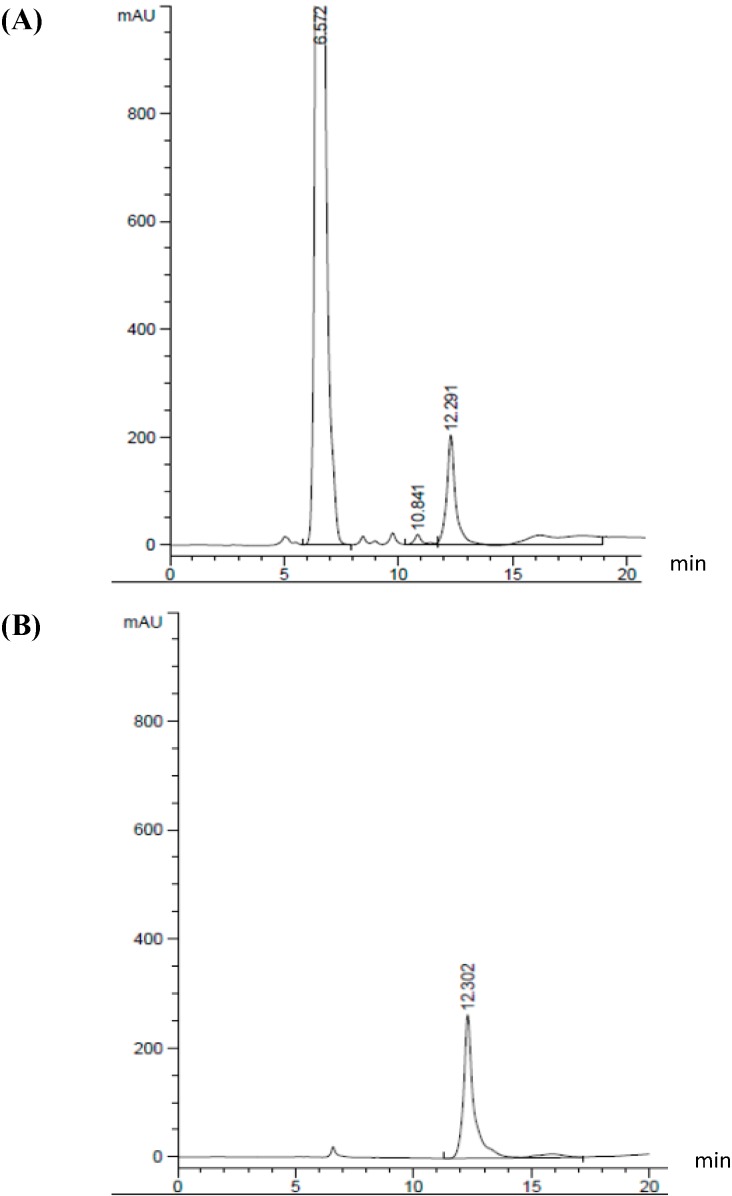
Representative chromatogram of GABA production using *Lactobacillus plantarum* Taj-Apis362 (**A**) and standard GABA (**B**).

The HPLC chromatograms of a GABA standard and one of the samples are shown in Figure 2. The mean GABA retention time was 12.291 ± 0.011 min. GABA has a suitable resolution (>0.5 min) from all the other amino acids [18]. In addition, calibration curves were obtained based on eight concentrations of GABA standard. The coefficient of determination (R^2^) was >0.9997. This is the first study to report on the evaluation of GABA-producing LAB originally isolated from honey stomachs and honeycombs of honeybees. Previous studies have isolated and evaluated GABA-producing LAB from traditional paocai [19], cheese and dairy products [8], traditional fermented fish [9], fish intestine [5] and fermented kimchi products [4,20].

### 2.2. Characterization of Lactobacillus plantarum Taj-Apis362

Cells are rod-shaped, 2.5–4 μm in length and 1–1.1 μm wide, Gram-positive, catalase-negative, non-spore-forming and non-motile. Gas is produced from glucose. Arginine dihydrolase and haemolyse were negative. Acid is produced from glucose, galactose, L-arabinose, fructose, maltose, mannitol, ribose, trehalose, melibiose, sorbitol, melezitose, lactose, mannose, esculin, cellobiose, salicin and sacchrose. Negative for acid production from amygdalin, inositol, dulcitol, raffinose and xylose. The overall results of the general identification and 16S rDNA sequencing [21] allowed us to assign that strain Taj-Apis362 DSM 13600 with a GenBank accession number of HM027644 belonged to the *Lactobacillus plantarum*.

### 2.3. Single Parameter

#### 2.3.1. Effect of Culture Temperature on Growth Profile and GABA Production

**Figure 3:**
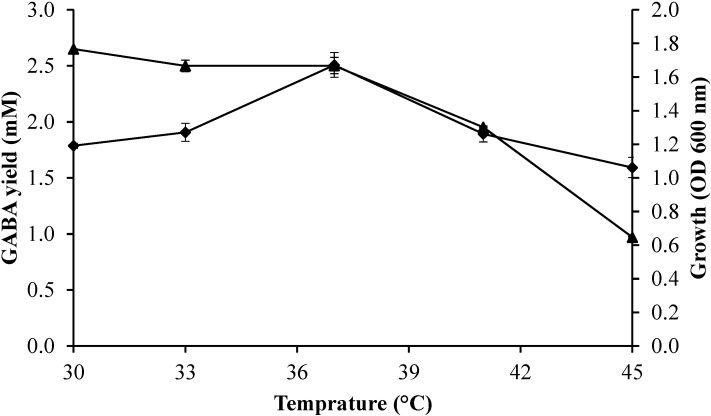
Effect of temperature on growth profile and GABA production by *Lactobacillus plantarum* Taj-Apis362. Culture conditions were fixed as follows: initial glutamic acid concentration, 50 mM; initial pH, 5; and incubation time, 60 h. Symbols: (▲) growth; (♦) GABA. The vertical bars represent the standard deviations (SD) from 3 replicates.

The effect of culture temperature from 30 to 45 °C on the bacterial growth profile and GABA production was determined using fixed fermentation parameters (initial glutamic acid concentration of 50 mM; initial pH of 5; incubation time of 60 h) in the culture medium. Figure 3 shows enhancement of GABA concentration with increasing the culture temperature from 30 to 37 °C, where maximum GABA produced was obtained, followed by a reduction of GABA production when the culture temperature exceeded 37 °C. The bacterial growth was peaked at culture temperature of 30 °C, and then decreased with the increase of culture temperature. *Lactobacillus plantarum* Taj-Apis362 produced low concentration of GABA at 45 °C, although the strain could grow under this fermentation temperature. Similarly, Li *et al.* [15] reported *Lactobacillus brevis* NCL912 growth increased with increased temperature and peaked at 35 °C, then decreased over the temperature. Moreover, *Lactobacillus* *plantarum* DSM19463 produced the highest GABA amounts between 30 °C and 35 °C [22]. In addition, Komatsuzaki *et al.* [9] demonstrated *Lactobacillus paracasei* NFRI 7415 displayed the highest of GABA-production at 37 °C.

#### 2.3.2. Effect of Initial pH of the Culture Medium on Growth Profile and GABA Production

The effect of initial pH from 4 to 7 on the bacterial growth profile and GABA production was determined using fixed fermentation parameters (initial glutamic acid concentration of 50 mM; culture temperature of 30 °C; incubation time of 60 h) in the culture mediumFigure 4 shows the enhancement of GABA concentration and biomass with increasing initial pH from 4 to 5.5, where the maximum GABA production and biomass were obtained, followed by a reduction of GABA production and cell growth when the initial pH exceeded 5.5. Moreover, *L. plantarum* Taj-Apis362 produced a low amount of GABA at initial pH 4. Similarly, Cho *et al*. [20] and Ko *et al*. [5] reported that GABA production by LAB decreased considerably at initial pH 4.0. A study conducted by Komatsuzaki *et al*. [23] demonstrated an optimal pH value for maintaining the activity of LAB GADs [24], and the high or low pH may lead to partial loss of GAD activity. This suggests that initial pH of 5 to 5.5 was more favorable for the production of GABA by *L. plantarum*.

**Figure 4:**
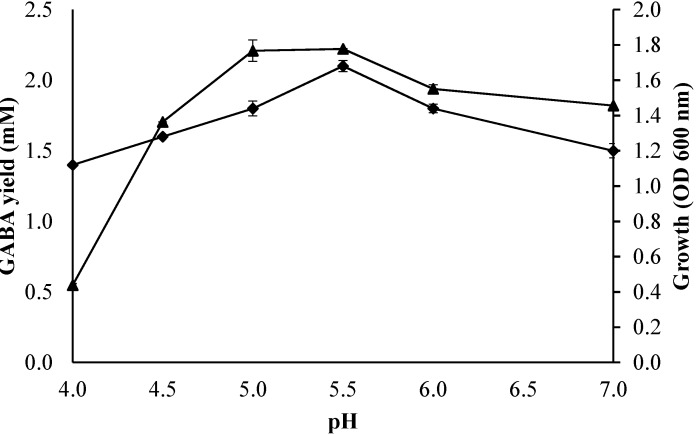
Effect of initial pH on growth profile and GABA production by *Lactobacillus plantarum*Taj-Apis362. Culture conditions were fixed as follows: initial glutamic acid concentration, 50 mM; culture temperature, 30 °C; and incubation time, 60 h. Symbols: (▲) growth; (♦) GABA. The vertical bars represent the SD from 3 replicates.

#### 2.3.3. Effect of Initial Glutamic Acid Concentrations on Growth Profile and GABA Production

**Figure 5:**
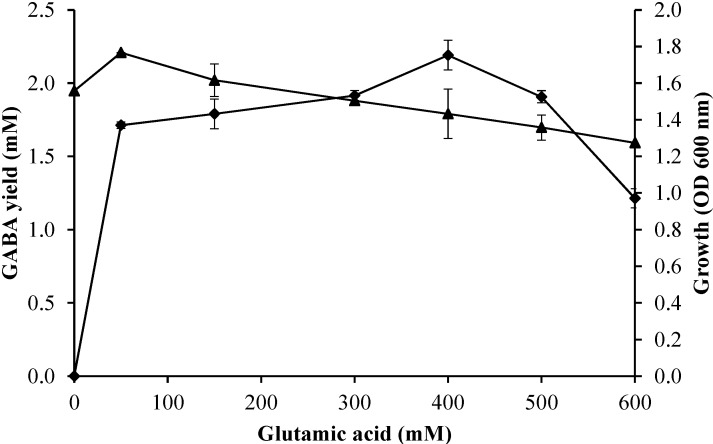
Effect of initial glutamic acid concentration on growth profile and GABA production of *Lactobacillus plantarum*Taj-Apis362. Culture conditions were fixed as follows: pH, 5; temperature, 30 °C; and incubation time, 60 h. Symbols: (▲) growth; (♦) GABA. The vertical bars represent the SD from 3 replicates.

The effect of initial concentrations of glutamic acid from 0 to 600 mM on the bacterial growth profile and GABA production was determined using fixed fermentation parameters (initial pH of 5; culture temperature of 30 °C; incubation time of 60 h) in the culture medium. Figure 5 shows the enhancement of GABA production with increasing the initial concentration of glutamic acid from 50 to 400 mM where the maximum GABA yield was obtained, followed by reduction of GABA production when the initial concentration of glutamic acid exceeded 600 mM. It was clear that too high a concentration of glutamic acid suppressed GABA production.

On the other hand, a high biomass was obtained with 50 mM initial glutamic acid. The bacterial growth decreased with the increase of initial glutamic acid concentration in the range of 100–600 mM. It is evident that a high concentration of glutamic acid suppressed the bacterial growth. Li *et al*. [25] reported the cell growth and biomass decreased with the increase of glutamate concentration at the given levels (0.25, 0.5, 0.75 and 1.0 M).

#### 2.3.4. Effect of Incubation Time on Growth Profile and GABA Production

**Figure 6:**
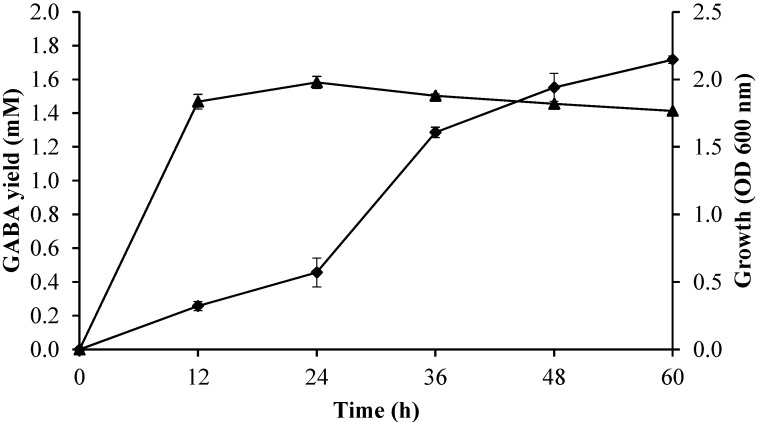
Effect of incubation time on growth profile and GABA production by *Lactobacillus* *plantarum* Taj-Apis362. Culture conditions were fixed as follows: initial glutamic acid concentration, 50 mM; temperature, 30 °C; and pH, 5. Symbols: (▲) growth; (♦) GABA. The vertical bars represent the S.D from three replicates.

The effect of incubation time from 0 to 60 h on the bacterial growth profile and GABA production was determined using fixed fermentation parameters (initial pH of 5; culture temperature of 30 °C; initial glutamic acid concentration of 50 mM) in the culture medium. As shown in Figure 6, the GABA production and biomass increased rapidly during the first 12 h of incubation and then increased slowly up to 60 h of incubation. The decrease in GABA biosynthesis could be due to the combined inhibitory effect of high concentration of GABA and glutamic acid. A similar observation was also reported by Li *et al*. [26].

The incubation time plays an important role in the GABA production. Cagno *et al*. [22] and Kim *et al*. [4] reported that the grape must and black raspberry juice fermented with *L. plantarum* DSM19463 and *L. brevis* GABA 100 reached the highest production of GABA, at 72 h and 15th day of the incubation time, respectively.

### 2.4. Analysis of Response Surface Methodology (RSM)

In order to model the fermentation process based on single variable optimization, the initial glutamic acid concentration, culture temperature, initial pH, and incubation time were chosen as effective variables in the response surface design in which the initial glutamic acid concentration of 525 mM, culture temperature of 37.5 °C, initial pH of 5.25, and 48 h incubated time were fixed as the central point for response surface analysis as shown in Table 1.

**Table 1:** *Lactobacillus plantarum* Taj-Apis362 treatment incorporations and responses.

Trials	Factor A Temperature (°C)	Factor B pH	Factor C Glutamic Acid (mM)	Factor D Time (h)	Actual GABA (mM)	Predicted GABA (mM)	Absolute Deviation
1	33.75	4.875	462.5	36	4.198 ± 0.004	4.283	0.0202
2	41.25	4.875	462.5	36	5.914 ± 0.017	5.769	0.0245
3	33.75	5.625	462.5	36	5.087 ± 0.023	5.103	0.0031
4	41.25	5.625	462.5	36	4.937 ± 0.015	5.137	0.0405
5	33.75	4.875	587.5	36	4.697 ± 0.002	4.632	0.0138
6	41.25	4.875	587.5	36	3.811 ± 0.006	4.067	0.0672
7	33.75	5.625	587.5	36	5.325 ± 0.004	5.131	0.0364
8	41.25	5.625	587.5	36	3.302 ± 0.002	3.113	0.0572
9	33.75	4.875	462.5	60	5.351 ± 0.004	5.261	0.0168
10	41.25	4.875	462.5	60	5.517 ± 0.022	5.720	0.0368
11	33.75	5.625	462.5	60	6.766 ± 0.004	6.520	0.0364
12	41.25	5.625	462.5	60	5.741 ± 0.005	5.527	0.0373
13	33.75	4.875	587.5	60	5.952 ± 0.001	5.762	0.0319
14	41.25	4.875	587.5	60	4.466 ± 0.009	4.170	0.0663
15	33.75	5.625	587.5	60	6.833 ± 0.004	6.698	0.0198
16	41.25	5.625	587.5	60	3.730 ± 0.050	3.655	0.0201
17	30.0	5.25	525	48	5.495 ± 0.005	5.770	0.0500
18	45.0	5.25	525	48	4.218 ± 0.005	4.212	0.0014
19	37.5	4.50	525	48	3.897 ± 0.004	3.882	0.0038
20	37.5	6.00	525	48	3.903 ± 0.008	4.187	0.0728
21	37.5	5.25	400	48	5.632 ± 0.001	5.593	0.0069
22	37.5	5.25	650	48	3.762 ± 0.007	4.070	0.0819
23	37.5	5.25	525	24	5.638 ± 0.003	5.521	0.0208
24	37.5	5.25	525	72	6.653 ± 0.009	7.040	0.0582
25	37.5	5.25	525	48	6.422 ± 0.003	6.674	0.0392
26	37.5	5.25	525	48	6.693 ± 0.004	6.674	0.0028
27	37.5	5.25	525	48	6.883 ± 0.001	6.674	0.0304
28	37.5	5.25	525	48	6.618 ± 0.002	6.674	0.0085
29	37.5	5.25	525	48	6.607 ± 0.004	6.674	0.0101
30	37.5	5.25	525	48	6.818 ± 0.004	6.674	0.0211

Notes: AAD = 3.1206%, R^2^ = 0.97. Values are means of three replicates ± standard deviation.

#### 2.4.1. Response Surface Methodology

Fitting the data to various models (linear, two factorial, quadratic and cubic) and their subsequent ANOVA (Table 2) showed that quadratic model (Equation (1)) was found to be the best model to explain the effects of effective factors on the GABA production.

**[GABA]** = 6.67 − 0.39A + 0.076B − 0.38C + 0.38D − 0.36AB − 0.51AC − 0.26AD − 0.08BC + 0.11BD + 0.038CD − 0.42A^2^ − 0.66B^2^ − 0.46C^2^ − 0.098D^2^ (1)

Where A is culture temperature, B is initial pH, C is initial glutamic acid concentration and D is incubation time.

**Table 2:** Analysis of variance (ANOVA) for the regression.

Source	SS	DF	MS	*F* Value	Prob ˃ *F*
Model	36.49708	14	2.606934	36.62527	<0.0001	significant
A	3.641286	1	3.641286	51.15706	<0.0001
B	0.139047	1	0.139047	1.953495	0.1825
C	3.477903	1	3.477903	48.86166	<0.0001
D	3.462349	1	3.462349	48.64314	<0.0001
AB	2.109061	1	2.109061	29.63056	<0.0001
AC	4.206169	1	4.206169	59.09320	<0.0001
AD	1.053595	1	1.053595	14.80214	<0.0016
BC	0.103610	1	0.103610	1.455634	0.2463
BD	0.192077	1	0.192077	2.698520	0.1212
CD	0.022921	1	0.022921	0.322022	0.5788
A^2^	4.853184	1	4.853184	68.18323	<0.0001
B^2^	11.94087	1	11.94087	167.7594	<0.0001
C^2^	5.816387	1	5.816387	81.71543	<0.0001
D^2^	0.265332	1	0.265332	3.727700	0.0726
Residual	1.067679	15	0.071179
Lack of Fit	0.931735	10	0.093173	3.426908	0.0932	not significant
Pure Error	0.135944	5	0.027189
Cor Total	37.56476	29

Notes: A, culture temperature (°C); B, initial pH; C, initial glutamic acid (mM); D, incubation time (h).

With very small “model *p*-value” (<0.0001) and not-significant “lack of fit” (*p*-value of 0.0932) from the analysis of ANOVA and a suitable coefficient of determination (R^2^ = 0.97) and adjusted coefficient of determination (R^2^_adjusted_ = 0.94), the quadratic polynomial model was highly significant and sufficient to represent the actual relationship between the response and the significant variables.

**Figure 7:**
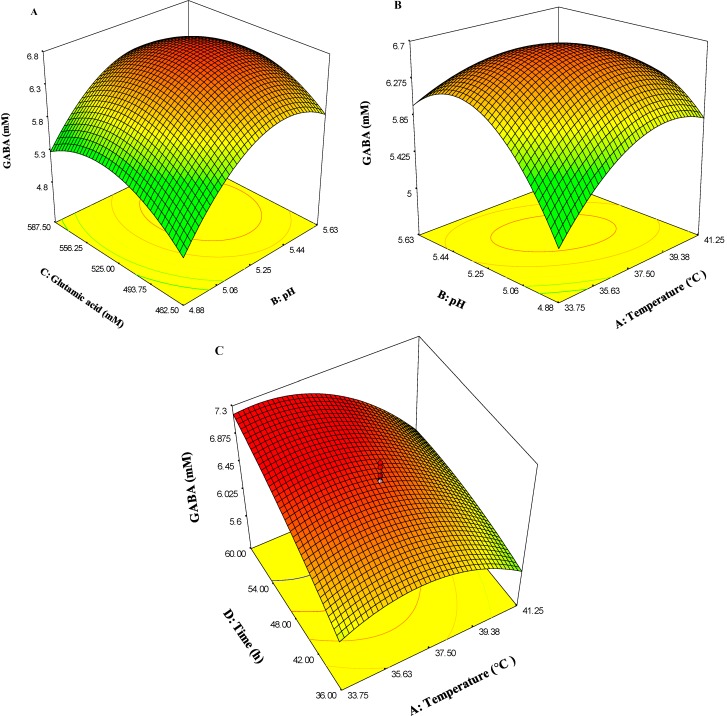
Three-dimensional surface plots showing the effect of different variables on GABA production. (**A**) Effect of initial glutamic acid and pH; (**B**) Effect of temperature and pH; (**C**) Effect of temperature and time.

The optimum level of each variable and the effect of their interactions on GABA production as a function of two variables were studied by plotting three-dimensional response surface curves (while keeping the other variables at central point). ANOVA analysis (Table 2) and three-dimensional plots (Figure 7) reveal that growth temperature; initial glutamic acid concentration and incubation period (time) had significant effects on GABA production. ANOVA analysis shows that although initial medium pH was not a significant parameter (*p* value > 0.05), it had important and significant interactions with other parameters; hence it has been used to develop the model. Figure 7 depicts that GABA production effectively increased with the increase in initial pH, culture temperature, initial glutamic acid concentration and incubation time until a certain value, followed by a decrease after that maximum value. On the other hand, ANOVA analysis reveals that temperature with *F*-value of 51.157 and *p*-value of <0.0001 is one of the most important parameters for GABA production.

Figure 7A shows the effect of initial glutamic acid and pH on the GABA production, where the value of culture temperature and incubation time were fixed at central point (37.5 °C, 48 h), respectively. As shown in the figure, GABA production increased with increasing amount of initial glutamic acid and increasing pH value, while the amount of initial glutamic acid and initial pH were at 513 mM and 5.33, respectively. Moreover, Figure 7A indicates that the initial pH with *F*-value of 1.953 exerted a slight effect on GABA yield and initial glutamic acid with *F*-value of 48.86 exerted a great effect.

Figure 7B shows the effects of culture temperature and initial pH value on the GABA production where the value of initial glutamic acid and incubation time were fixed at the central point (525 mM, 48 h). As shown in this Figure, the GABA increased with increasing culture temperature and increasing of pH value, while the culture temperature and initial pH were at the 35.5 and 5.33, respectively. Furthermore, Figure 7B indicates that the initial pH with *F*-value of 1.953 exerted a slight effect on GABA production and temperature with *F*-value of 51.157 exerted a great one.

Figure 7C shows the effect of culture temperature and incubation time on the GABA production, where the value of initial glutamic acid concentration and initial pH were fixed at central point (525 mM, 5.25) respectively. As shown in the Figure, the GABA increased with increased incubation time and culture temperature value, while the culture temperatures were around 35–36 °C. However, GABA production decreased if culture temperature was increased to 41.5 °C. It is apparent that high temperature was harmful to the GABA production. Moreover, Figure 7C indicates that the incubation time with *F*-value of 48.643 effects and culture temperature with *F*-value of 51.157 exerted a great effect on GABA production.

#### 2.4.2. Verification of the Fitted Model and Optimum Point

In order to verify the model, the actual values of GABA production of *L. plantarum* Taj-Apis362 was compared to the predicted values by calculation the AAD (Table 3). The calculated AAD for this quadratic model was 1.505% which indicated that the model equation was accurate and highly reliable. The predicted optimum condition; the factor levels were set at the optimal values given by the quadratic equation using Design Expert software. The optimal conditions for GABA production were predicted as presented in Table 3 along with their predicted and actual values. The optimum conditions for the highest GABA production (7.21 mM) were obtained at culture temperature of 36 °C, initial glutamic acid of 497.97 mM, initial pH of 5.31, and incubation time of 60 h. The experimental value of 7.15 mM was very close to the predicted value of 7.21 mM.

**Table 3:** Optimum condition solutions.

No.	Temperature (°C)	pH	Glutamic Acid (mM)	Time (h)	Actual GABA (mM)	Predicted GABA (mM)	Absolute Deviation
1	36	5.31	497.97	60	7.15 ± 0.015	7.210	0.0084
2	37	5.16	462.50	60	6.94 ± 0.024	6.842	0.0141
3	37.5	5.31	514.88	48	6.57 ± 0.009	6.730	0.0243
4	37.5	5.33	462.50	48	6.48 ± 0.018	6.567	0.0134

Notes: R^2^, 0.8436; AAD, 1.5072%. Values are means of three replicates ± standard deviation.

## 3. Experimental Section

### 3.1. Isolation and Identification of GABA-Producing LAB

*Lactobacillus* strains were isolated and identified previously from honeycomb and honey stomach of the Asiatic giant honeybee (*A. dorsata*) in Malaysia [11,12,21].

### 3.2. Culture Medium and Conditions

Lactobacilli MRS broth (Merck, Darmstadt, Germany) was autoclaved at 118 °C for 15 min and used for GABA production and maintenance of *Lactobacillus* strains. The LAB strains were incubated in 10 mL MRS broth in universal bottles at 30 °C, without shaking. The inoculation size was 1% with approximately 8 logs CFU/mL. Glutamic acid (Merck) was dissolved in distilled water, autoclaved separately and added after sterilization of MRS broth.

### 3.3. Evaluation of GABA-Producing LAB

A total of 24 dominant strains of LAB isolated in the previous study [11,12,21] were evaluated for their ability to produce GABA. All strains were grown in MRS medium (pH 5) containing 50 mM of glutamatic acid (Merck) for 60 h at 30 °C. GABA content in the supernatants was measured.

### 3.4. Measurement of GABA Content

GABA content was determined by an Agilent 1200 series HPLC system (Agilent Tech, Waldron, Germany) equipped with a Hypersil ODS C18 reverse-phase column with 5 μm diameter, 250 mm length and 4.6 mm internal diameter (Thermo Fisher Scientific Co., Waltham, MA, USA). A 100-μL culture broth filtered through a 0.22-μm filter, was derivatized and the residue was dissolved in 20 μL of an ethanol-water-triethylamine (2:2:1) solution and evaporated by the vacuum pump at 300 millitorr. Thirty μL of an ethanol–water–triethylamine–phenylisothiocyanate solution (7:1:1:1) was added into a tube and incubated at room temperature for 20 min to allow the formation of phenylisothiocyanate-GABA and vacuumed again at 300 millitorr. After derivatization, the sample was diluted and subjected to HPLC analysis. The injection volume was 20 μL with a flow rate of 0.6 mL/min.

The HPLC mobile phase A was a mixture: Sodium acetate three hydrates (10.254 g, 99%, A.C.S. reagent, Sigma-Aldrich, Saint Louis MO, USA) dissolved in 900 mL deionized water and 500 μL trimethylamine (Merck), which was made up to one liter with deionized water. The pH of the mobile phase A was adjusted to 5.8 using glacial acetic acid (Merck). HPLC mobile phase B was acetonitrile (HPLC grade, Merck) and mobile phase C was deionized water. All mobile phases were passed through a 0.22 μm membrane filter. The column temperature was set up at 25 °C; sample injection volume was 20 µL and the compound was detected through a diode array detector at 254 nm. The amount of GABA was calculated by comparing the peak area with the corresponding GABA standard.

### 3.5. Characterization of Lactobacillus plantarum Taj-Apis362

The colony morphology was investigated on MRS agar after 48 h of incubation at 37 °C under anaerobic conditions. Conventional biochemical tests (e.g., partial sequence analysis of the 16S rDNA, the analysis of the cellular fatty acids and differentiating individual phenotypic tests) were performed at the DSMZ (Deutsche Sammlung von Mikroorganismen und Zellkulturen, Braunschweig, Germany) on the *L. plantarum* Taj-Apis362. Growth characteristics were determined in MRS broth. *Lactobacillus* *plantarum* Taj-Apis362 grew at 15 °C and 45 °C.

### 3.6. Single Parameter Optimization

The purpose of the preliminary step was to identify the range of the fermentation parameters that had a significant effect on GABA production within the ranges under study. Single variable optimization was carried out in order to analyze the influence of four fermentation parameters, including initial glutamic acid concentration (0–600 mM), culture temperature (30–45 °C), initial pH (4–7) and incubation time (0–60 h) on GABA production by *L. plantarum* Taj-Apis362.

### 3.7. Experimental Design

A five-levels-four-variables-central composite design (CCD) was employed in this study, resulting in 30 combinations (Table 1). Culture temperature (30–45 °C), initial pH (4.5–6), incubation time (24–72 h) and initial glutamic acid concentration (400–650 mM) were the independent factors selected to optimize the GABA production by *L. plantarum* Taj-Apis362. To avoid bias, 30 treatments were performed in a random order in which 24 axial points (treatment 1–24) and six center points (treatment 25–30) were considered (Table 1). Each experiment was performed in triplicate.

### 3.8. Response Surface Methodology (RSM)

The CCD design experimental data were used for model fitting in RSM to find the best polynomial equation. These data were analyzed using interpreted Design Expert version 7.0 trial software (Stat Ease Inc., Minneapolis, MN, USA). Three main analytical steps involving analysis of variance (ANOVA), a regression analysis and the plotting of response surface were performed to establish an optimum condition for GABA production. Then, the predicted values obtained from RSM model, were compared with actual values for testing the model. Finally, the experimental values of predicted optimal conditions were used as validating set and were compared with predicted values.

### 3.9. Verification of Estimated Data

To test the estimation capabilities of the technique, the estimated responses obtained from RSM were compared with the observed responses using the coefficient of determination (R^2^) and absolute average deviation (AAD). The R^2^ and AAD are calculated by following equations:

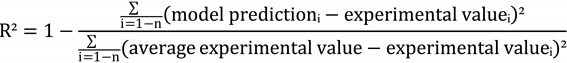
(2)
where the *n* is the number of experimental data.


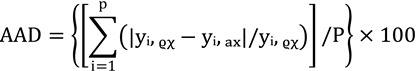
(3)
where *y_i_*_,e*x*_ and *y_i_*_,ax_ are the experimental and calculated responses, respectively, and *p* is the number of the experimental run.

## 4. Conclusions

In conclusion, to our knowledge, this study is the first to evaluate the GABA producing LAB obtained from the honey stomachs and honey combs of honeybees. In this study 24 *Lactobacillus* strains that had been isolated from the honey stomachs and honeycombs of honeybees were evaluated for their GABA-producing ability. Out of 24 LAB strains, Taj-Apis362 showed the highest GABA-producing capability (1.76 mM) in MRS broth containing 50 mM initial glutamic acid cultured during 60 h incubation. The effects of culture temperature, incubation time, initial pH and initial glutamic acid concentration on the GABA production by *L. plantarum* Taj-Apis362 with one-variable-at-a-time experiments were further investigated. Culture temperature, initial glutamic acid concentration and incubation time had a significant effect on GABA production by *L. plantarum* Taj-Apis362 was greatly enhanced by using RSM and reached 7.15 mM, which was 2.86-fold higher than that of one-variable-at-a-time experiments.

In addition, the initial pH in culture medium changes with incubation time during fermentation, hence, the initial pH influenced final biomass and GABA-production. This discovery of *Lactobacillus* with the ability to synthesize GABA may offer new opportunities in the design of improved health promoting functional foods, with the benefits of enriched GABA and probiotic bacteria. Such strains will accelerate the development of functional fermented foods. However, further study is needed to develop a recombinant Lb-GAD for maximum GABA production.
